# 644. Impact of COVID-19 on Tuberculosis Control and Surveillance: An 11-Year Retrospective Analysis in Belo Horizonte, Brazil

**DOI:** 10.1093/ofid/ofae631.209

**Published:** 2025-01-29

**Authors:** Marco Aurélio Angelo, Débora De Vasconcelos, Giovana Ferreira, Meritxell Bassas, Laiz Almeida, Walisson Ferreira Carvalho, Bráulio R G M Couto

**Affiliations:** Hospital Risoleta Tolentino Neves - HRTN, Belo Horizonte, Minas Gerais, Brazil; Hospital Risoleta Tolentino Neves - HRTN, Belo Horizonte, Minas Gerais, Brazil; Hospital Risoleta Tolentino Neves - HRTN, Belo Horizonte, Minas Gerais, Brazil; Hospital Risoleta Tolentino Neves - HRTN, Belo Horizonte, Minas Gerais, Brazil; Hospital Risoleta Tolentino Neves - HRTN, Belo Horizonte, Minas Gerais, Brazil; PUC MInas, Belo Horizonte, Minas Gerais, Brazil; AMECI – Associação Mineira de Epidemiologia e Controle de Infecções, Belo Horizonte, Minas Gerais, Brazil

## Abstract

**Background:**

Non-pharmacological measures to control the spread of COVID-19 imposed severe restrictions on citizens' mobility and prioritized access to health equipment for serious and emergency cases. These measures limited contact opportunities for patients within tuberculosis surveillance systems, reduced access to antimicrobial therapy, and increased the risk of disease transmission during mandatory confinements, exposing vulnerable populations. Studies indicate that, both in Brazil and globally, the COVID-19 pandemic has significantly set back the fight against TB, affecting early diagnosis and access to health equipment. This setback is reflected in Brazil's first increase in TB mortality since 2005. We present the TB incidence over eleven years (2013-2023), analyzing factors associated to TB cure in a public reference hospital in Belo Horizonte, Brazil.

**Figure 1:**
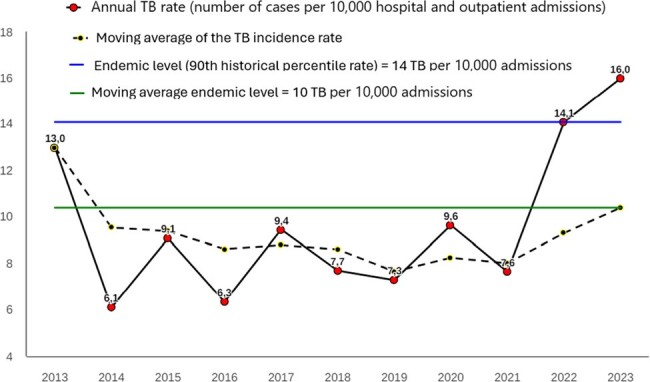
Incidence Rate of Respiratory Tuberculosis (2013-2023) at a Reference Public Hospital in Belo Horizonte, Brazil: The incidence nearly doubled in the post-pandemic period (2022-2023) compared to the historical period, with rates rising from 8.6 to approximately 15 cases per 10,000 hospital and outpatient admissions.

Incidence Rate of Respiratory Tuberculosis (2013-2023) at a Reference Public Hospital in Belo Horizonte, Brazil: The incidence nearly doubled in the post-pandemic period (2022-2023) compared to the historical period, with rates rising from 8.6 to approximately 15 cases per 10,000 hospital and outpatient admissions.

**Methods:**

Retrospective observational study to conduct TB surveillance over an 11-year period (Jan/2013 to Dec/2023). Data were collected from a public reference hospital in Belo Horizonte city, Brazil. Monthly incidence rates were calculated using the number of TB cases as the numerator and the total number of hospital and outpatient admissions as the denominator. Univariate and multivariate analyses were conducted to identify factors associated with TB cure.

**Figure 2:**
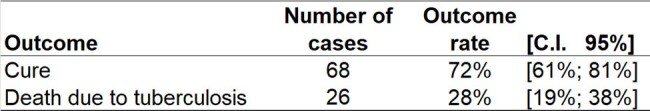
Probability of TB Cure and Risk of Death: Excluding TB patients who abandoned treatment, were transferred to other hospitals, or are still under treatment, the analysis shows a 72% probability of TB cure.

Probability of TB Cure and Risk of Death: Excluding TB patients who abandoned treatment, were transferred to other hospitals, or are still under treatment, the analysis shows a 72% probability of TB cure.

**Results:**

From 2013 to 2021, TB incidence was stable; however, after 2021, there was a subtle but noticeable increase in incidence (Fig. 1). A sample of 94 TB patients that has a complete defined outcome, i.e., cure or death due to TB, was retrieved from the database: 68 was completed cured (72%; Fig. 2). In the multivariate analysis, four factors were associated with TB cure: Age , Patient from Belo Horizonte city (capital of Minas Gerais State), New case of tuberculosis, and Directly Observed Treatment Administered (Fig. 3). The ROC analysis of the logistic model demonstrated good predictive accuracy (Fig. 4).

**Figure 3:**
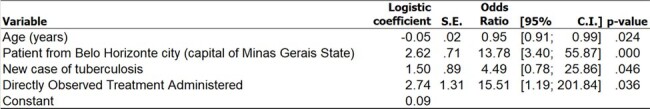
Logistic Regression Model of TB Treatment Success: Age as a Risk Factor for Treatment Failure, while Residence in the State Capital (Belo Horizonte), Being a New (Acute) TB Case, and Receiving Directly Observed Treatment are Protective Factors for TB Cure.

Logistic Regression Model of TB Treatment Success: Age as a Risk Factor for Treatment Failure, while Residence in the State Capital (Belo Horizonte), Being a New (Acute) TB Case, and Receiving Directly Observed Treatment are Protective Factors for TB Cure.

**Conclusion:**

The micro-level analysis of TB cases at a single public hospital likely reflects the broader trends observed across the state of Minas Gerais. The post-COVID era presents significant challenges for managing numerous diseases, particularly tuberculosis. Implementing Directly Observed Treatmen is essential for achieving successful outcomes in TB treatment.

**Figure 4:**
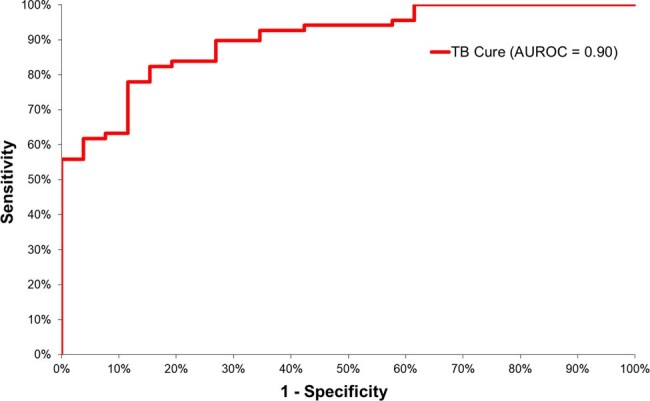
ROC Analysis of the Logistic Model: Area Under the ROC Curve = 0.90, Indicating Good Predictive Accuracy.

ROC Analysis of the Logistic Model: Area Under the ROC Curve = 0.90, Indicating Good Predictive Accuracy.

**Disclosures:**

**All Authors**: No reported disclosures

